# Scientific Items

**Published:** 1888-01

**Authors:** 


### SCIENTIFIC ITEMS.

**Professor Tyndall on Lightning Conductors**—Professor Tyndall, writing to the London *Times*, says: "Your recent remarks on thunderstorms and their effects induce me to submit to you the following facts and considerations. Some years ago a rock lighthouse on the coast of Ireland was struck and damaged by lightning. An engineer was sent down to report the occurrence, and as I then held the honorable post of scientific adviser to the Trinity House and Board of Trade, the report was submitted to me. The lightning conductor had been carried down the lighthouse tower, its lower extremity being carefully embedded in a stone, perforated to receive it. If the object had been to invite the lightning to strike the tower, a better arrangement could hardly have been adopted. I gave directions to have the conductor immediately prolonged, and to have added to it a large terminal plate of copper, which was to be completely submerged in the sea. The obvious convenience of a chain as a prolongation of the conductor caused the authorities in Ireland to propose it, but I was obliged to veto the adoption of the chain. The contact of link with link is never perfect. I had, moreover, beside me a portion of a chain cable through which a lightning discharge had passed, the electricity in passing from link to link encountering a resistance sufficient to enable it to partially fuse the chain. The abolition of resistance is absolutely necessary in connecting a lightning conductor with the earth, and this is done by closely embedding in earth a plate of good conducting material and of large area. The largeness of area makes atonement for the imperfect conductivity of earth. The plate, in fact, constitutes a wide door through which the electricity passes freely into the earth, its disruptive and damaging effects being thereby avoided. These truths are elementary, but they are often neglected. I watched with interest sometime ago the operation of setting up a lightning conductor on the house of a neighbor of mine in the country. The wire rope, which formed part of the conductor, was carried down the wall, and comfortably laid in the earth below, without any terminal plate whatever. I expostulated with the man who did the work, but he obviously thought he knew more about the matter than I did. I am creditably informed that this is a common way of dealing with lightning conductors by ignorant practitioners, and the Bishop of Winchester's palace at Farnham has been mentioned to me as an edifice 'protected' in this fashion. If my informant is correct, the 'protection' is a mockery, a delusion, and a snare."—*Scientific American.*

**A Sea Telephone.**—A dispatch from Cincinnati says: At Fernback, ten miles from this city, are the workshop and laboratory of Harvey B. Cox, a young electrician, who, though known to but few here, is attracting the attention of scientists and electricians in this country and in Europe by his inventions, in which he is as prolific and ingenious as Edison. His latest device is a trumpet to be used for telephoning at sea, on which he has been working for some months. This invention is the outgrowth of his discovery of the great distance an echoed or reverberated sound will carry and the discovery that speaking trumpets, if made to give the same fundamental note, would vibrate and produce the phenomenon known in acoustics as "sympathy." With this trumpet, conversation in an ordinary tone of voice was carried on between persons four and a quarter miles apart. People sitting at their windows or on their porches a mile away conversing in an ordinary tone could be distinctly heard, and in a couple of instances they were told the nature of their conversation and admitted that such had taken place. By listening to the whistle of a train, and tracing it to and beyond Farnbank to Lawrenceburg, Ind., it was found that the instrument has a well-defined range of twenty-five miles ; that is a loud sound like a locomotive whistle or the rumbling of train can be distinctly heard at a distance of thirteen miles in every direction. Conversation was readily carried on between two gentlemen on high hills on opposite sides of the Ohio river, about four and one-half miles apart. Tests made on the water showed that the trumpet was even more available than on land. The instrument will be patented as soon as perfected. A name has not yet been chosen for it. Mr. Cox has a great many other curious and valuable devices, both electrical and mechanical, but none as curious as his telephone.—*Scientific American.*

**A Remakarble Case of Amnesia.**—The many strange phenomena of amnesia have been enriched by the experience of one of the ablest living pcychologists, Professor Bain. Some months ago Professor Bain fell from his horse, and was unconscious for about three hours afterward. During this time his shoulder, which had been sprained by the accident, was set without his knowledge. Upon regaining consciousness, it was found that he had lost all remembrance of what had occurred an hour before the accident, as well as of three hours following. He was found on a different road from that which he can remember having intended to take, and so must have changed his mind. Of this he has lost all recollection ; otherwise there were no mental effects. The editor of *Mind*, who tel s the story, adds another case in which a gentleman, after falling from a carriage, remained unconscious for nearly four months. Upon re-awakening, not only was this interval a total blank to him, but the events of the week preceding the accident were equally lost. Important transactions which he had made during the week were forgotten This suggests that there may be some relation between the duration of unconsciousness after the accident and the memory blank before. At all events, the phenomena, mysterious as they are, deserve to be recorded. The authenticity and careful analysis of the above cases add to their value.

